# Genetic Determinants of Telomere Length and Their Role in Human Disease: Molecular Mechanisms and Underrepresented Populations’ Perspectives

**DOI:** 10.3390/biomedicines14061211

**Published:** 2026-05-27

**Authors:** Viviana A. Ruiz-Pozo, Santiago Cadena-Ullauri, Rafael Tamayo-Trujillo, Patricia Guevara-Ramírez, Elius Paz-Cruz, Alejandro Cabrera-Andrade, Ana Karina Zambrano

**Affiliations:** 1 Universidad UTE, Facultad de Ciencias de la Salud Eugenio Espejo, Centro de Investigación Genética y Genómica, Quito 170129, Ecuador; viviana.ruiz@ute.edu.ec (V.A.R.-P.); santiagoa.cadena@ute.edu.ec (S.C.-U.); victor.tamayo@ute.edu.ec (R.T.-T.); patyguevara28@gmail.com (P.G.-R.); elius.paz@ute.edu.ec (E.P.-C.); 2 Grupo de Bio-Quimioinformática, Universidad de Las Américas, Quito 170125, Ecuador; raul.cabrera@udla.edu.ec; 3 Carrera de Enfermería, Facultad de Ciencias de la Salud, Universidad de Las Américas, Quito 170125, Ecuador

**Keywords:** telomeres, disease, genetics, genomics, healthcare

## Abstract

Telomere length (TL) is a key determinant of cellular aging and genomic stability, influenced by genetic, molecular, and environmental factors. Progressive telomere shortening has been associated with degenerative and cardiovascular diseases, whereas longer telomeres have been linked to an increased risk of cancer, highlighting a dual and context-dependent relationship between TL and disease susceptibility. Evidence from genome-wide association studies (GWAS) and Mendelian randomization analyses indicates that TL is a highly heritable and polygenic trait, regulated by variants in genes such as *TERT*, *TERC*, *RTEL1*, and *POT1*, as well as components of the shelterin and CST complexes. This study integrates genetic variants associated with telomere shortening and elongation, including their functional classification, proposed molecular mechanisms, and ACMG/AMP categorization, together with global and Latin American allele frequency data. These variants may participate in key processes such as telomerase activity, telomerase RNA stability, and telomere replication, thereby influencing susceptibility to multiple diseases. However, current evidence is largely derived from European and Asian populations. Given the highly admixed nature of Latin American populations, population-specific studies are required to identify unique genetic determinants and to improve the application of precision medicine.

## 1. Introduction

Cellular mechanisms and intercellular interactions are tightly regulated processes that can be influenced by intrinsic and extrinsic cues [1,2,3]. Alterations in these processes are associated with progressive telomere shortening, a central hallmark of cellular aging and senescence [1,4,5]. Telomeres are non-coding DNA regions mainly composed of tandem 5’-TTAGGG-3’ repeats and their complementary 3’-AATCCC-5’ strands, which can fold into t-loop structures at chromosome termini [6,7]. These structures maintain chromosomal integrity by preventing aberrant end-to-end fusions, supporting DNA replication, and acting as a molecular clock that limits cellular proliferation [8,9]. Due to the end-replication problem inherent to DNA polymerase, telomeres progressively shorten by 50 to 150 base pairs during each cell division in most somatic cells. This is because DNA polymerases require RNA primers and cannot fully replicate the terminal region of the lagging strand after primer removal, leading, after successive cell divisions, to telomere attrition [10,11,12]. This mechanism provides a molecular basis for Hayflick’s limit, although telomerase activity or alternative lengthening of telomeres can delay or bypass this process in specific cell types [8,13,14,15].

In contrast, cells with telomerase activity, such as germline cells, stem cells, and many cancer cells, can partially or fully counteract this shortening, thereby maintaining telomere length and proliferative capacity [16].

Given their role in genome protection, dysregulation of telomere maintenance mechanisms can affect cellular homeostasis and has been associated with numerous pathological processes, including neurodegeneration, hematopoietic failure, premature aging disorders, oncogenesis, and tumor progression [8,17]. Furthermore, telomere length (TL) shows a dual relationship with disease risk, in which shorter telomeres could increase susceptibility to degenerative, fibrotic, and cardiovascular diseases, whereas longer telomeres may lead to an elevated risk of various types of cancer, potentially due to an extended proliferative window before senescence [18,19,20].

TL is a highly heritable trait influenced by environmental factors, polygenic inheritance, and both common and rare genetic variants [21,22]. Individuals may inherit relatively long or short telomeres from their parents, and even parent-of-origin effects have been reported [23,24]. Genome-wide association studies (GWAS) have been historically used to identify numerous loci influencing TL, including *TERT*, *TERC*, *RTEL1*, *POT1*, *DKC1*, *NOP10*, *NHP2*, *GAR1*, and CST complex genes, supporting that TL behaves as a classic quantitative trait shaped by multiple gene–gene interactions [1,3,9,22,25].

Despite rapid advances in telomere genetics, most studies have been conducted in individuals of European ancestry, resulting in a substantial knowledge gap in our understanding of TL variation and telomere-related disease risk in underrepresented populations [24,26,27]. For instance, Latin American populations, characterized by a complex admixture of Native American, European, and African ancestries [28], remain markedly underrepresented in genomic studies. Thus, it is of the utmost importance to analyze this genetic diversity, as it could influence the distribution and functional impact of TL-associated variants [24,26,27].

The aim of this review is to describe genetic variants linked to telomere shortening and elongation, evaluating their relationship with disease risk and potential functional mechanisms and highlighting the distribution and frequency differences in these variants in Latin American populations.

## 2. The Telomere—Telomerase Maintenance System

**Figure 1:**
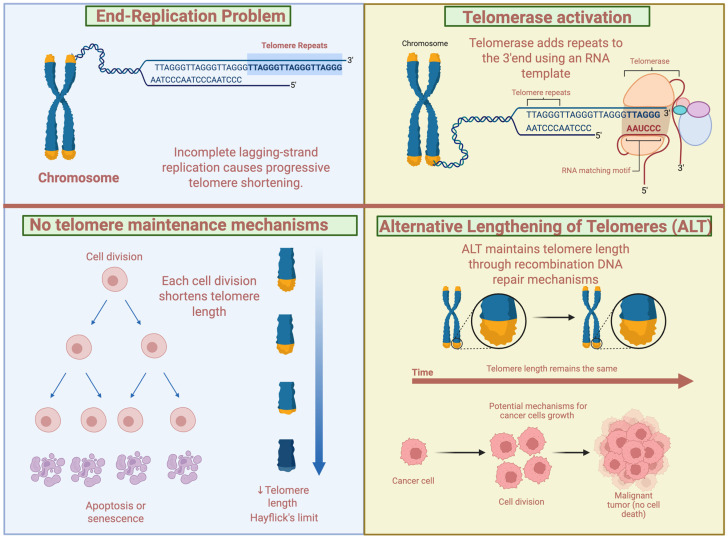
End-replication problem and telomere maintenance mechanisms. The figure summarizes the contrasting outcomes of telomere attrition and telomere maintenance. In cells lacking active telomere maintenance mechanisms, incomplete lagging-strand replication causes progressive telomere shortening after successive cell divisions. When telomeres become critically short, chromosome-end protection is compromised, triggering cellular senescence or apoptosis and contributing to Hayflick’s limit. In contrast, telomere maintenance can occur through telomerase activation, in which telomerase adds telomeric repeats to the 3’ end using its RNA template, or through alternative lengthening of telomeres (ALT), a telomerase-independent, recombination-based mechanism. Sustained telomere maintenance can preserve proliferative capacity and, in cancer cells, may contribute to continued tumor growth. Created in BioRender. Ruiz-Pozo, V.A. (2026) https://BioRender.com/62w7rn1 (accessed on 7 May 2026).

TL homeostasis is regulated by a coordinated molecular network composed of the telomerase ribonucleoprotein complex, telomere-binding protein assemblies, and accessory factors that regulate DNA replication and chromosome-end protection (Figure 1). Disruptions in the components of these systems can lead to telomere dysfunction, genomic instability, and altered susceptibility to a wide spectrum of human diseases [29,30].

Telomerase is a ribonucleoprotein enzyme that compensates for the progressive loss of telomeric DNA that occurs during conventional DNA replication. Its catalytic activity relies on two essential core components: telomerase reverse transcriptase (hTERT), which mediates nucleotide addition, and the telomerase RNA component (hTERC), which provides the template for *de novo* synthesis of telomeric repeats at the 3′ chromosome terminus. Together, these components enable the extension of telomeres in proliferative cell types [8,31,32].

Telomere elongation can also be achieved through alternative lengthening of telomeres (ALT), a telomerase-independent mechanism observed in approximately 5–15% of human cancers [11,33,34]. ALT relies on homology-directed DNA repair and break-induced replication-like processes, in which telomeric DNA is extended using homologous telomeric sequences as templates [11]. This mechanism is characterized by marked telomere length heterogeneity, telomeric recombination, extrachromosomal telomeric repeats, ALT-associated promyelocytic leukemia bodies, and increased chromosomal instability [33,34]. Although ALT can occur in several tumor types, it is particularly enriched in cancers of mesenchymal or neuroepithelial origin and is frequently associated with alterations in chromatin-remodeling and telomere-associated genes, especially *ATRX* and *DAXX*, as well as *H3F3A*, *SMARCAL1*, and *SLX4IP* [11]. Given its role in telomerase-negative tumors, ALT has prognostic relevance in selected cancers and represents a potential therapeutic target [11].

Additional protein complexes participate in telomere synthesis and maintenance. For instance, the dyskerin ribonucleoprotein subcomplex, composed of *DKC1*, *NOP10*, *NHP2*, and *GAR1*, stabilizes telomerase assembly and promotes its association with telomeric DNA [35,36,37]. This complex binds to hTERC, protecting it from degradation and facilitating its incorporation into functional telomerase [35,36]. Genetic variants affecting dyskerin complex components are well-established contributors to telomere biology disorders (TBDs), underscoring the importance of telomerase biogenesis in TL regulation [38,39,40].

Telomere integrity is further safeguarded by the shelterin complex, a six-protein assembly composed of the telomeric repeat-binding factors TERF1 and TERF2, the adaptor proteins TIN2 and TPP1 (encoded by *ACD*), the single-stranded telomeric DNA-binding protein POT1, and RAP1 (encoded by *TERF2IP*) [9,41]. Shelterin regulates telomere maintenance through two mechanisms. First, it promotes the formation of higher-order telomeric structures, including the t-loop, preventing inappropriate activation of DNA damage response pathways. Second, shelterin modulates telomerase accessibility and activity at chromosome ends [6,9]. In particular, the POT1–TPP1 complex modulates telomerase recruitment and telomere maintenance [42,43]. Variants in shelterin genes can therefore exert pleiotropic effects by influencing processes such as telomere capping and elongation dynamics [6,9,43].

The shelterin complex also interacts with accessory proteins for telomere protection, including SNM1B [44,45]. This protein is a TRF2-associated exonuclease that localizes to telomeres through direct interaction with TRF2 and contributes to post-replicative telomere processing by facilitating the generation of the 3′ single-stranded G-rich overhang. Pathogenic variants in *SNM1B* have been associated with dyskeratosis congenita–spectrum telomeropathies, highlighting its key role in maintaining telomere integrity and genome stability [44,45].

Following telomerase-mediated extension of the G-rich strand, the CST complex (CTC1, STN1, and TEN1) promotes the recruitment of the DNA polymerase α-primase complex to the telomeric tail, enabling the complementary C-strand fill-in synthesis and restoring double-stranded telomeric DNA at chromosome ends, while preserving the 3′ single-stranded G-rich overhang required for proper telomere capping and t-loop formation [9,46]. Disruption of CST function results in defective telomere replication, telomere fragility, or excessive telomere elongation, highlighting its role in telomerase maintenance [46,47,48,49].

Various accessory proteins also participate in telomere length regulation by modulating DNA replication, chromatin structure, and telomere architecture [50,51]. For example, *RTEL1* encodes a DNA helicase that unwinds t-loop structures during replication, preventing telomere fragility and aberrant recombination events [50,51]. Loss-of-function variants in *RTEL1* are associated with telomere shortening and telomere syndromes [50,51,52].

Similarly, variants in *ZNF208* have been associated with differences in telomere length, potentially influencing telomere maintenance through transcriptional or chromatin-mediated mechanisms [53,54,55]. Emerging evidence also suggests a role for *DCAF4* in telomere biology, although its mechanistic contribution remains incompletely understood [2,56].

In addition to TL regulation, correct processing of chromosome ends is essential for maintaining telomere integrity. After DNA replication, telomeres must generate a 3′ single-stranded G-rich overhang, necessary for t-loop formation and capping. Disruption of this process can trigger DNA damage responses and genomic instability, even without evident telomere shortening, highlighting that defects in end processing may represent an important and independent mechanism underlying TBDs [25,45,57,58]. For instance, the Apollo exonuclease contributes to post-replicative telomere processing and 3′ overhang formation, and variants in *SNM1B* have been linked to severe TBDs with clinical features resembling dyskeratosis congenita and Hoyeraal–Hreidarsson syndrome, despite normal overall telomere length [45]. These findings can indicate that telomere dysfunction can arise from impaired structural processing rather than telomere shortening alone.

Beyond inherited genetic variation, telomere length is also influenced by epigenetic regulation and by environmental, psychosocial, and lifestyle exposures. In fact, telomere length itself can be considered an epigenetic trait because it is not fully determined by the genome alone; rather, it is shaped by the physical length of the telomeres inherited from parents [22,59]. DNA methylation and histone modifications at telomeric and subtelomeric regions can influence telomere chromatin structure, stability, and maintenance. At the same time, several external factors including smoking, chronic stress, obesity, poor sleep, unhealthy dietary patterns, and socioeconomic adversity have been associated with shorter telomeres [60], whereas regular physical activity and healthier diets may help preserve telomere length [61]. Moreover, studies suggest that stress could reduce leukocyte telomere length in affected individuals and contribute to transgenerational epigenetic inheritance; for instance, maternal stress can result in the transmission of shorter telomeres to offspring [62]. This inherited telomere shortening may contribute to genetic anticipation, in which disease onset occurs earlier and phenotypes become more severe across successive generations [22].

Collectively, these molecular components form an integrated network, and genetic variation across these components can disrupt telomere length dynamics, replication stability, and ultimately cellular lifespan. Importantly, population-specific variants may influence telomere maintenance genes, leading to ancestry-dependent differences in telomere length and disease susceptibility, highlighting the importance of conducting population-specific research.

## 3. Methods for Telomere Length Measurement

The main techniques for measuring TL range from traditional laboratory “gold standards” to high-throughput epidemiological tools and advanced single-molecule sequencing methods [63,64,65]. These protocols may differ in technical requirements, resolution, throughput, and clinical applicability. Some approaches estimate average telomere length across all chromosomes, whereas others provide information about individual chromosome ends, specific cell populations, or the shortest telomeres, which may be particularly relevant for cellular senescence and TBDs [63,64,65].

### 3.1. Terminal Restriction Fragment (TRF) Analysis

TRF analysis has historically been considered the gold-standard method for measuring TL because it estimates absolute mean TL in kilobases. It is based on restriction enzyme digestion of genomic DNA, followed by agarose gel electrophoresis and Southern blotting with telomere-specific probes. However, this method requires relatively large amounts of high-quality DNA, is labor-intensive, and mainly provides an average TL estimate without detecting the shortest telomeres [25,63,64,65].

### 3.2. Quantitative Polymerase Chain Reaction (qPCR)

qPCR is one of the most widely used methods in large epidemiological and population-based studies. It estimates relative TL by calculating the telomere-to-single-copy gene ratio, known as the T/S ratio. Its main advantages are that it is rapid, inexpensive, high-throughput, and requires only small amounts of DNA. However, qPCR usually provides a relative rather than an absolute TL estimate and is sensitive to DNA quality, assay conditions, and inter-assay variability [25,63,64,65,66].

### 3.3. Fluorescence In Situ Hybridization (FISH)-Based

FISH-based approaches use fluorescently labeled telomeric probes to quantify telomere signals at the cellular or chromosomal level. Quantitative FISH (Q-FISH) can estimate TL at individual chromosome ends, making it useful for chromosome-specific studies and for identifying telomere-free ends. Flow-FISH combines FISH with flow cytometry and allows TL measurement in specific leukocyte subsets, making it particularly useful for the clinical evaluation of telomere biology disorders [25,63,64,65,67,68].

### 3.4. Single Telomere Length Analysis (STELA), Universal STELA, and TeSLA

High-resolution methods such as STELA, Universal STELA, and the Telomere Shortest Length Assay (TeSLA) are designed to assess individual or critically short telomeres. STELA uses a ligation-based PCR approach to amplify telomeres from specific chromosome ends, whereas Universal STELA can detect critically short telomeres regardless of chromosomal location. TeSLA provides sensitive measurement of the shortest telomeres across chromosomes, which may be especially relevant for studies of cellular senescence and aging [25,63,64,65,67,68].

### 3.5. Sequencing-Based Bioinformatic Approaches

Sequencing-based methods estimate TL from whole-genome or whole-exome sequencing data using computational tools such as TelSeq or Computel, which quantify reads containing telomeric repeats and normalize them against genomic reads. These approaches are useful for large-scale biobank and genomic studies because they allow TL estimation from existing sequencing datasets. In addition, long-read sequencing platforms, such as Oxford Nanopore and PacBio HiFi, may enable chromosome-specific telomere assessment at higher resolution [11,12,63,64,69].

## 4. Telomere Length and Human Disease Risk

Telomere length is a key indicator of cellular aging and genomic stability, as telomeres are progressively lost in most somatic cells during replication due to the end-replication problem, whereby removal of the terminal RNA primer leaves the chromosome end incompletely replicated [10,11,12]. Over successive cell divisions, critically short telomeres can compromise chromosome-end protection, activate DNA damage responses, and induce cellular senescence or apoptosis, providing a molecular basis for Hayflick’s limit [14,15].

**Figure 2:**
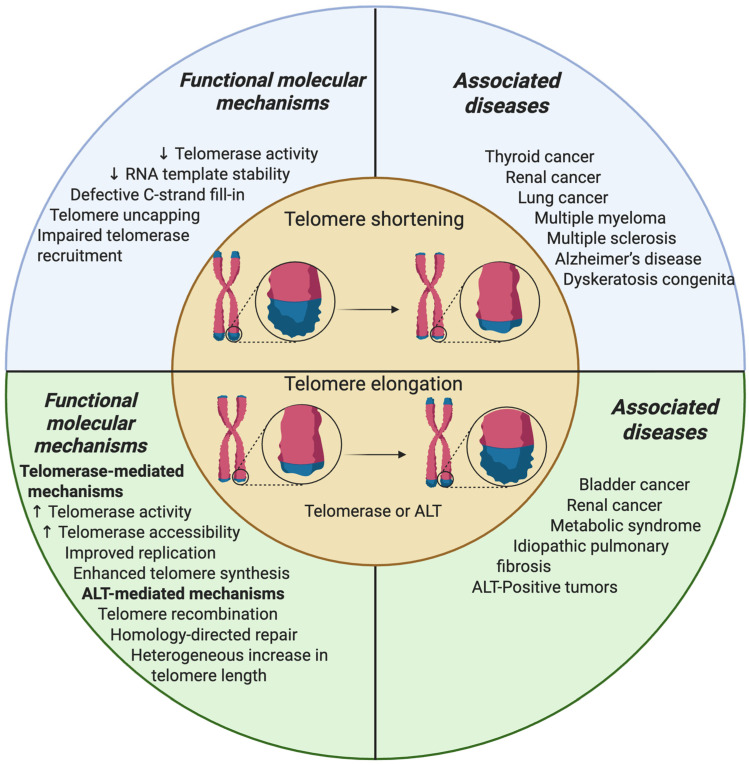
Telomere length dynamics and disease associations. Schematic representation of telomere shortening (top) and telomere elongation (bottom), highlighting their opposing roles in telomere homeostasis and their differential associations with cancer, cardiovascular and metabolic diseases, neurodegenerative diseases, and telomere biology disorders (TBDs). Created in BioRender. Ruiz-Pozo, V.A. (2026) https://BioRender.com/b7esf17 (accessed on 7 May 2026).

Emerging evidence has shown that TL is associated with a bidirectional relationship with human disease risk, as both shortened and elongated telomeres have been linked to different disorders [70]. Dysregulated longer telomeres have been correlated with an increased risk of several malignancies, including glioma and thyroid, kidney, and lung cancers [71]. In contrast, abnormally shorter telomeres are more frequently associated with cardiovascular, neurodegenerative, and pulmonary diseases (Figure 2) [72].

### 4.1. Cancer

Longer telomeres are associated with a higher risk of certain types of cancer. One study included 420,081 cases and 1,093,105 controls, predominantly of European ancestry. The analyzed cohort encompassed a wide range of phenotypes, including 35 cancer types, such as glioma, ovarian, lung, melanoma, neuroblastoma, bladder, kidney, and endometrial cancers, as well as 48 non-neoplastic diseases, including cardiovascular, autoimmune, and pulmonary disorders [73]. Diseases were classified into primary and secondary outcomes according to the study’s statistical criteria (power threshold > 50% for detecting associations), and statistical models were used to estimate the odds ratio for disease risk per genetically determined increase in TL. The results showed that increases in TL were correlated with a higher risk in 9 of the 22 primary cancers analyzed. Notably, telomere elongation was also associated with a reduced risk in 6 of the 32 primary non-neoplastic diseases evaluated. These findings suggest that longer telomeres may support tissue maintenance and reduce susceptibility to some age-related degenerative conditions, while also increasing the risk of several malignancies by extending cellular replicative capacity and delaying senescence [73].

In a Mendelian randomization study, GWAS data from East Asian populations were used to assess the relationship between TL and cancer risk. Genetic variants (SNPs) associated with TL were used as instrumental variables. The exposure dataset for TL included 16,759 individuals from the Singapore Chinese Health Study, whereas the outcome datasets for cancer incidence included the Korean Cancer Prevention Study-II (KCPS-II, *n* = 159,844), the Korean Genome Epidemiology Study (KoGES, *n* = 211,285), and the Biobank of Japan (BBJ, *n* = 201,800) [74]. Overall, the results showed that longer TL was associated with a 1.36-fold increased risk of developing cancer. The strongest associations were observed for thyroid cancer (2.50-fold increased risk), kidney cancer (2.43-fold), and lung cancer (1.83-fold). Stratification by histological subtype further showed that lung adenocarcinoma exhibited a stronger association (3.83-fold) at the localized stage than at distant or regional stages. These findings suggest that longer TL may contribute to the initiation and early development of lung cancer; however, it does not appear to determine disease progression [74].

Notably, this association between longer TL and lung adenocarcinoma risk is concordant with findings from the European multi-cancer study, where longer TL was associated with increased risk across 9 of 22 primary cancers [73], despite differences in population ancestry, supporting cross-ancestry consistency of this association.

In a case–control study of a Hispanic Caucasian population comprising 1385 cases and 1385 controls, TL showed subtype-specific associations in lung cancer. Longer telomeres were associated with an increased risk of lung adenocarcinoma (*n* = 706), particularly in women and individuals younger than 60 years. This association between longer TL and increased cancer risk is similar to that reported in the European [73] and East Asian [74] cohorts described above. In contrast, longer telomeres were associated with a reduced risk of squamous cell carcinoma (*n* = 320), especially in men, representing a clear histological divergence not observed in the other two cohorts and underscoring the importance of subtype stratification when comparing findings across study designs [75].

In multiple myeloma, a Mendelian randomization study based on genetic data from European and Asian populations, including 2407 cases and 1741 controls, showed that a greater genetic predisposition to long telomeres, assessed using a teloscore based on 11 SNPs, increased disease risk (OR = 1.69), but also correlated with better overall survival (HR = 0.93), suggesting that TL may influence susceptibility and prognosis through distinct mechanisms. These findings demonstrate that long telomere length could act as a dual factor, influencing both disease susceptibility and, paradoxically, improving survival among affected patients [76].

Conversely, shorter leukocyte TL has been linked to an increased risk of certain types of cancer. A study analyzed TL in peripheral blood leukocytes to identify a prognostic biomarker in three cohorts: 144 patients with bladder cancer, 144 with renal cell carcinoma, and 73 individuals in the control group (with no history or diagnosis of malignant diseases). The results demonstrated that patients with bladder cancer and renal cell carcinoma had significantly shorter telomeres than controls. Furthermore, multivariate analysis identified short telomeres as an independent predictor of decreased overall survival in bladder cancer (*p* = 0.039) and renal cell carcinoma (*p* = 0.041). Notably, these findings refer to prognosis in patients with established cancer rather than to cancer susceptibility, suggesting that the biological mechanisms linking TL to survival outcomes may differ from those involved in incident cancer risk [77].

In another study using 18,430 samples from 9127 patients across 31 cancer types available through The Cancer Genome Atlas, it was observed that 70% of tumors have shorter TL than normal tissue. However, mutations in *TERT* were associated with *ATRX* gene inactivation [78]. Among these mechanisms, alternative lengthening of telomeres (ALT) represents a recombination-based telomere maintenance pathway. ALT is frequently associated with ATRX or DAXX inactivation, telomeric chromatin dysregulation that generates replicative stress and promotes ALT activation, the formation of ALT-associated subnuclear promyelocytic leukemia (PML) bodies, extrachromosomal telomeric repeats, and break-induced replication-mediated telomere extension [79]. Furthermore, pan-cancer studies based on 11,123 samples across 33 cancer types indicate that telomere maintenance mechanisms are heterogeneous, including telomerase-dependent, ALT-dependent, mixed, and low-activity profiles, underscoring the diversity and context-dependent regulation of telomere maintenance strategies across cancer types. Importantly, this study evaluated the activities of telomerase-dependent (TEL) and ALT pathways and observed intermediate-to-high activity in both ALT and TEL pathways in 31–40% of cancers. Taken together, these findings highlight that telomere maintenance in cancer is not limited to telomerase activation but also includes ALT and other heterogeneous TMM mechanisms with potential biological and prognostic relevance [80]. Collectively, these findings highlight the diversity of telomere maintenance strategies in cancer [78,81].

### 4.2. Cardiovascular and Metabolic Diseases

Multiple observational and cohort studies demonstrate an association between TL and cardiovascular and metabolic diseases, suggesting that telomere shortening may be a key mechanism of cellular senescence that contributes to both biological aging and the development and progression of these pathologies [82].

A cohort based on the NHANES database included 1980 participants from the United States with metabolic syndrome (MetS). The results indicated that individuals with MetS and shorter TL had a higher risk of all-cause mortality (HR = 1.33) and cardiovascular mortality (HR = 1.36) than those with longer TL after a follow-up of 17.75 years [83]. Similarly, a large-scale analysis of 7252 adults evaluated the association between leukocyte TL and 17 cardiovascular biomarkers and found that shorter TL was associated with increased adiposity, systemic inflammation (as measured by C-reactive protein), dyslipidemia, and elevated blood pressure [84]. Another study including 1556 adults with high-risk clinical profiles characterized by obesity, hyperglycemia, hypertension, and dyslipidemia, found that these individuals had shorter TL compared with those in the low-risk group [85]. These findings suggest that metabolic and cardiovascular abnormalities are associated with accelerated biological aging and telomere shortening.

In addition, Mendelian randomization analyses based on 52 independent genetic variants associated with telomere length, derived from a GWAS of 78,592 individuals of European ancestry, indicated that genetically longer TL was associated with an increased risk of MetS (OR = 1.133), as well as with greater abdominal adiposity and hypertension [86]. Similarly, a GWAS-based genomic analysis evaluating the relationship between leukocyte TL and cardiovascular disease identified an overlap of 248 genetic loci, including *ALDH2*, *ACAD10*, and *SH2B3*. These shared loci may independently influence both TL and cardiovascular disease susceptibility, suggesting that TL may not be exclusively a biomarker of metabolic disease risk [87].

Some studies further suggest that dysregulation of the neurohormonal axis contributes to heart disease, with increased expression of NOX2 (NADPH oxidase 2) and HDAC6 promoting reactive oxygen species (ROS) overproduction and severe oxidative stress. The relationship between oxidative stress and TL could be explained by the finding that elevated ROS levels can inhibit PRDX1, an antioxidant protein that protects telomeres. As a result, oxidative DNA damage and physical shortening of cardiomyocyte telomeres may occur, ultimately promoting the development of heart disease [88,89]. Furthermore, in vivo research suggests that mitochondrial dysfunction can contribute to telomere attrition in cardiovascular disease models. In a mutant murine model, partial SOD2 (a mitochondrial antioxidant) deficiency induced mitochondrial oxidative stress and progressive telomere shortening in aortic smooth muscle cells, despite increased telomerase activity. This effect was not observed in Sod1+/− mice (cytoplasmic antioxidant), indicating that mitochondrial, rather than cytoplasmic, oxidative stress is associated with telomere dysfunction and cardiovascular phenotypes [90].

### 4.3. Neurodegenerative Disorders

Population-based, genetic, and case–control studies have shown that TL plays a complex role in neurodegenerative disease susceptibility [91,92]. In a prospective UK Biobank cohort of 459,902 individuals, predominantly of European ancestry, shorter TL was associated with a higher risk of Alzheimer’s disease and dementia, whereas longer TL was correlated with an increased risk of multiple sclerosis (HR = 3.71). Furthermore, longer TL was also linked to a 48% decreased risk of Alzheimer’s disease [72]. These findings are supported by Mendelian randomization analyses demonstrating a causal and protective effect of long telomeres against Alzheimer’s and increased longevity [93]. Consistently, a study in an Italian population (*n* = 534) reported a reduction in TL in 255 patients with late-onset Alzheimer’s disease, including 120 sporadic and 135 familial cases, compared with 279 controls, independent of age, sex, and APOE-ε4 genotype. Taken together, these findings suggest that telomere shortening reflects biological aging and increased susceptibility to neurodegeneration, whereas longer TL may exert disease-specific effects [94].

Certain biological processes, particularly oxidative stress and inflammation, are associated with telomere shortening and may subsequently contribute to neurodegenerative disease onset. Reactive oxygen species generated by mitochondria can induce telomeric DNA damage, thereby promoting telomere shortening and cellular senescence. In addition, oxidative damage can trigger neuroinflammatory responses mediated by toxic protein accumulation, microglial dysfunction, and the release of pro-inflammatory cytokines, all of which are characteristic of disorders such as Parkinson’s disease and Alzheimer’s disease [95,96].

### 4.4. Telomere Biology Disorders

TBDs, also commonly referred to as telomeropathies, are a group of rare diseases caused by germline mutations affecting genes involved in telomere maintenance [97,98]. TBDs are characterized by deficient telomere maintenance, resulting in age-inappropriate telomere shortening [99]. TBDs comprise a group of multisystemic conditions with clinical manifestations ranging from childhood to adulthood, including dyskeratosis congenita (DC), idiopathic pulmonary fibrosis, and related disorders [100].

In autosomal dominant TBDs, genetic anticipation is a feature whereby affected offspring may present with earlier disease onset and more severe phenotypes than their parents [101]. Studies in DC and other related telomeropathies have associated this pattern with mutations in *TERT*, *TERC*, *TINF2*, *RTEL1*, *POT1*, and other telomere maintenance genes [102]. Moreover, anticipation is not explained by genotype alone, but by the inheritance of both the pathogenic variant and progressively shorter telomeres across generations [103].

Family-based clinical studies and cellular models have linked pathogenic mutations in *DKC1* to telomere dysfunction syndromes such as DC. DC is a genetic disorder characterized by shorter TL, leading to bone marrow failure and cancer predisposition [99]. A study analyzed two unrelated families and identified missense variants in *DKC1* (p.Thr49Ser and p.Pro409Arg) on the X chromosome by performing whole-exome sequencing. These mutations compromised the stability of the telomerase RNA component (TR), resulting in reduced RNA levels and impaired telomere maintenance [104]. Similarly, a study of pluripotent stem cells derived from patients harboring *DKC1* mutations (Q31E, A353V, and ΔL37) demonstrated reduced telomerase RNA levels, decreased telomerase activity, and significantly shortened telomeres. Targeted gene editing revealed that certain mutations, such as A353V, exert dominant-negative effects that hinder the correction of mutation-associated defects and their phenotypic consequences, whereas other variants may be partially responsive to functional restoration [105].

Consistently, a study of 61 patients, including 28 with idiopathic pulmonary fibrosis and 33 with other lung diseases, demonstrated significantly reduced survival among individuals with idiopathic pulmonary fibrosis compared with those with other pulmonary conditions. Notably, shorter telomere length was independently associated with decreased survival in this patient group (*p* = 0.085) [106].

## 5. Genetic Variants Determining Telomere Length

Telomere length is a quantitative trait influenced by multiple factors, including an estimated heritability ranging from 44% to 84% [22,25,68]. Variation in TL arises from both rare high-penetrance mutations and the cumulative effects of numerous common genetic variants [1,2,6]. While pathogenic variants in telomere maintenance and protection genes cause classical TBDs, interindividual variation in TL within the general population largely reflects a polygenic architecture involving regulatory, replication-associated, and genome-maintenance pathways [21,22,25,66,68,107].

GWAS have expanded our understanding of TL, revealing an integrated network composed of telomerase components, replication machinery, and DNA repair pathways [21,25,66,69,107,108,109]. In this regard, early studies identified variants in the core telomerase genes *TERT* and *TERC* as determinants of leukocyte telomere length (LTL) [3,69,110,111,112,113,114,115,116,117]. Subsequent GWAS supported the role of these loci and identified additional variants in genes involved in telomere replication and stability, such as *RTEL1*, *POT1*, *DKC1*, *NOP10*, and *CTC1* [3,111,118,119,120,121,122,123,124,125,126,127,128].

Moreover, large biobank-scale studies have now identified more than 150 independent loci associated with TL [69,107,109,118,129,130,131]. Many of these loci extend beyond canonical telomere biology genes and involve pathways related to DNA repair, chromatin regulation, replication timing, and oxidative stress responses, highlighting that TL regulation implicates a broad genome stability network [25,66,67,68,109,132,133,134]. Based on their effect on TL, genetic variants can be classified into those associated with telomere shortening or telomere elongation.

It is important to distinguish between clinical pathogenicity and quantitative effects on telomere length. ACMG/AMP classifications such as benign, likely benign, or variant of uncertain significance indicate that a variant is not currently established as a monogenic cause of disease, but they do not exclude a potential role in telomere length regulation [135,136]. Large-scale GWAS have shown that many TL-associated variants exert modest, polygenic effects and are in or near genes involved in telomere biology, including *TERT* and *TERC*, which encode the catalytic and RNA components of telomerase, as well as *RTEL1*, *POT1*, and genes of the shelterin and CST complexes [109,137,138,139,140].

These variants may influence TL through regulatory mechanisms affecting gene expression, telomerase formation or activity, telomerase recruitment, telomere replication efficiency, C-strand fill-in, DNA damage responses, or telomere homeostasis under environmental or replicative stress [137,138,139,140]. Therefore, non-deleterious variants listed in Table 1 and Table 2 should be interpreted as potential TL-modifying alleles rather than directly pathogenic variants [136,137,140,141]. Their effects may be context-dependent and shaped by polygenic inheritance, linkage disequilibrium with causal variants, ancestry-specific allele frequencies, and gene–environment interactions [109,137,138,139]. Importantly, recent fine-mapping studies have shown that variants classified as benign or likely benign may still be statistically associated with disease risk, although their effects are moderate and do not correspond to Mendelian disease-causing effect sizes [136].

### 5.1. Genetic Variants Associated with Telomere Shortening

Genetic variants associated with reduced TL primarily affect telomerase activity, RNA template stability, and telomere replication efficiency, C-strand fill-in synthesis, and chromosome-end processing (Table 1) [67,68,116,142]. Pathogenic and regulatory variants in *TERT* have been linked to shortened telomeres and disease susceptibility. Representative variants include rs199422294, rs770066110, rs1554038048, rs121918661, rs114616103, and rs34052286, which have been correlated with dyskeratosis congenita, pulmonary fibrosis, melanoma, and leukemia [3,40,69,109,110,111,112,113,114,115,116,117]. Additional variants, such as rs2853677, have also been linked to non-small cell lung cancer and leukemia [113,114], while rs2735940 has been associated with coronary artery disease [108,115].

Variants in *TERC*, including rs1553915617 and rs12638862, may reduce telomerase RNA stability and availability, contributing to telomere shortening and disease susceptibility. These variants have been associated with dyskeratosis congenita, pulmonary fibrosis, and hematologic malignancies such as chronic lymphocytic leukemia, Hodgkin lymphoma, and multiple myeloma [3,40,69,109,143,144]. Similarly, variants in the helicase gene *RTEL1*, including rs41309367 and rs3787089, have also been correlated with dyskeratosis congenita, pulmonary fibrosis, leukemia, and Hoyeraal–Hreidarsson syndrome, which could reflect impaired resolution of telomeric secondary structures during DNA replication [3,40,69,109,118,119,145].

Variants affecting the shelterin complex gene *POT1*, including rs113394869, rs587777475, rs143635917, rs947005337, and rs1385542313, may disrupt telomere protection and have been linked to tumor-predisposition syndromes, pulmonary fibrosis, thyroid carcinoma, melanoma, and chronic lymphocytic leukemia [40,69,109,111,120,121,124]. Furthermore, variants in *DKC1* (rs146700772, rs1557264102) and *NOP10* (rs121908092) have been associated with dyskeratosis congenita and multisystem manifestations, such as cataracts, hearing impairment, nephrotic syndrome, and enterocolitis [3,30,37,40,69,109,125,126,128].

Lastly, variants in the CST complex, including *CTC1* (rs3027234) and *STN1* (rs10883948, rs9420907), may impair telomere replication completion by disrupting C-strand fill-in synthesis. These alterations have been associated with disorders such as cerebroretinal microangiopathy and Coats plus syndrome, as well as malignancies including pancreatic, colorectal, and neuroendocrine cancers [40,69,109,131,146,147,148,149,150].

**Table 1:** Genetic variants associated with telomere shortening: Functional classification, mechanisms, and population frequencies.

Telomere Effect	Gene	Variant ID	Variant Type	Functional Category	Proposed Mechanism	AMCG/AMP Classification	Global MAF	Latin American MAF	References
Telomere length shortening	*TERT*	rs199422294	Missense	Telomerase catalytic subunit	Reduced telomerase activity	Pathogenic	<0.01%	Not reported	[3,40,69,110,151,152,153]
		rs770066110	Missense	Telomerase catalytic subunit	Impaired telomerase function	Pathogenic	<0.01%	Not reported	[3,40,69,151,152,153]
		rs1554038048	Missense	Telomerase catalytic subunit	Telomerase instability	Likely pathogenic	Not reported	Not reported	[3,40,69,151,152,153]
		rs121918661	Missense	Telomerase catalytic subunit	Reduced enzymatic activity	Likely benign	<0.01%	0.2%	[40,69,111,112,151,152,153]
		rs2853677	Intronic regulatory	Telomerase expression	Reduced TERT transcription	Benign	~59%	~65%	[40,69,113,114,151,152,153]
		rs114616103	Intronic regulatory	Telomerase expression	Altered telomerase regulation	Benign	~2.9%	~1.3%	[40,69,151,152,153]
		rs34052286	Intronic regulatory	Telomerase expression	Regulatory effect on telomerase	Benign	~0.5%	~1.5%	[40,69,151,152,153]
		rs2735940	Regulatory	Telomerase catalytic subunit	TERT promoter regulation	Benign	~49%	~57%	[40,69,108,115,151,152,153]
		rs2736100	Regulatory	Telomerase catalytic subunit	Telomerase activity modulation	Benign	~50%	~56.5%	[40,69,116,117,151,152,153]
	*TERC*	rs1553915617	Rare variant	Telomerase catalytic subunit	Potential telomerase dysfunction	Likely pathogenic	Not reported	Not reported	[40,69,110,143,144,151,152,153]
		rs12638862	Intronic	Telomerase catalytic subunit	Transcriptional regulation	Benign	26.4%	33%	[40,69,110,151,152,153]
	*RTEL1*	rs41309367	Regulatory	Telomerase helicase	Altered telomerase replication	Benign	68.8%	69%	[40,69,110,145,151,152,153]
		rs3787089	Regulatory	Telomerase helicase	Replication-associated telomere instability	Benign	69.9%	70.5%	[40,69,110,118,119,151,152,153]
	*POT1*	rs113394869	Rare variant	Shelterin complex	Potential telomerase instability	Benign	16%	Not reported	[40,69,110,151,152,153]
		rs587777475	Rare variant	Shelterin complex	Telomerase dysfunction	VUS	<0.01%	Not reported	[40,69,110,120,121,122,151,152,153]
		rs143635917	Rare variant	Shelterin complex	Telomere shortening	VUS	0.05%	N/A	[40,69,110,121,151,152,153]
		rs947005337	Rare variant	Shelterin complex	Telomerase dysfunction	Likely pathogenic	<0.01%	N/A	[40,69,110,111,123,124,151,152,153]
		rs1385542313	Rare variant	Shelterin complex	Telomere instability	VUS	<0.01%	N/A	[40,69,110,111,151,152,153]
	*DKC1*	rs146700772	Rare variant	Telomerase RNA stabilization complex	Reduced telomerase activity	Benign	0.03%	0.01%	[40,69,110,125,126,127,151,152,153]
		rs1557264102	Rare variant	Telomerase RNA stabilization complex	Telomere shortening	Pathogenic	Not reported	Not reported	[3,40,69,110,128,151,152,153]
	*NOP10*	rs121908092	Missense	Telomerase RNA stabilization complex	Telomerase deficiency	VUS	<0.01%	Not reported	[37,40,69,151,152,153]
	*CTC1*	rs3027234	Intronic	CST complex	Telomere replication instability	Benign	19%	12%	[40,69,146,147,148,151,152,153]
	*STN1*	rs10883948	Regulatory	CST complex	Altered telomerase expression	Benign	35%	Not reported	[40,69,151,152,153]
		rs9420907	Intronic	CST complex regulator	Regulation of telomere replication and C-strand synthesis	Benign	82%	82%	[40,69,131,148,149,150,151,152,153]

### 5.2. Genetic Variants Associated with Telomere Elongation

Genetic variants associated with telomere elongation influence telomerase expression, replication efficiency, and telomerase accessibility to chromosome ends (Table 2) [38,67,107,109,142,154]. Variants in *TERT*, including rs7705526, rs2736100, rs6897196, and rs192999400, have been linked to increased telomerase activity and longer TL. Notably, these variants have also been correlated with an increased risk of melanoma, acute myeloid leukemia, pulmonary fibrosis, ovarian cancer, coronary artery disease, and Lynch syndrome, suggesting a potential association between telomere elongation and cancer susceptibility [40,69,107,109,118,131,155,156].

Variants in *TERC*, such as rs2293607, have been associated with pulmonary fibrosis and various types of cancer, including colorectal and bladder cancer [40,69,109,157,158,159], while a variant (rs61753459) in *RTEL1* has also been linked to telomere elongation and TBDs, indicating its role in maintaining replication efficiency at telomeric regions [40,69,109,160]. Variants in *POT1*, including rs202187871 and rs750470470, may increase telomerase accessibility to telomeres, contributing to telomere elongation while also increasing the risk of chronic lymphocytic leukemia and Hodgkin lymphoma [40,69,107,109,111,121,154,161].

Moreover, variants in *STN1*, including rs9420907, rs1265164, and rs111447985, may influence telomere replication dynamics and have been correlated with conditions such as Coats plus syndrome and uterine leiomyoma [40,69,109,162,163,164]. These variants act through pathways that enhance telomerase-mediated elongation or improve telomere replication stability, potentially promoting longer TL, which may lead to an increased oncogenic risk [38,67,107,109,142,154].

Overall, genetic variants can potentially modulate TL by interacting with multiple molecular pathways that control telomerase activity, telomere protection, replication fidelity, and genome stability, leading to alterations in TL and potentially influencing susceptibility to various diseases, including cancer.

**Table 2:** Genetic variants associated with telomere elongation: Functional classification, mechanisms, and population frequencies.

Telomere Effect	Gene	Variant ID	Variant Type	Functional Category	Proposed Mechanism	AMCG/AMP Classification	Global MAF	Latin American MAF	References
Telomere length elongation	*TERT*	rs7705526	Intronic regulatory	Telomerase catalytic subunit	Increased telomerase activity and telomere elongation	VUS	<0.01%	Not reported	[40,69,118,151,152,153,155,156]
		rs2736100	Intronic regulatory	Telomerase catalytic subunit	Modulation of telomerase activity	VUS	<0.01%	Not reported	[40,69,107,131,151,152,153]
		rs6897196	Intronic regulatory	Telomerase catalytic subunit	Regulation of TERT expression	Benign	43.7%	33.5%	[40,69,151,152,153]
		rs192999400	Rare variant	Telomerase catalytic subunit	Potential telomerase activity alteration	Benign	0.7%	0.35%	[40,69,151,152,153]
	*TERC*	rs12638862	Intronic regulatory	Telomerase RNA component	Transcriptional regulation	Benign	26.4%	33%	[40,69,130,143,144,151,152,153]
		rs2293607	Intronic regulatory	Telomerase RNA component	Increased telomerase RNA stability	VUS	Not reported	Not reported	[40,69,151,152,153,157,158,159]
	*RTEL1*	rs61753459	Rare variant	Telomerase helicase	Potential telomerase functional alteration	VUS	Not reported	Not reported	[40,69,151,152,153,160]
	*POT1*	rs202187871	Rare variant	Shelterin complex	Telomere elongation via altered telomerase activity	VUS	<0.01%	Not reported	[40,69,107,111,121,151,152,153,161]
		rs750470470	Rare variant	Shelterin complex	Potential regulatory effect on telomerase	Pathogenic	<0.01%	Not reported	[40,69,121,151,152,153,154]
	*STN1*	rs9420907	Intronic regulatory	CST complex regulator	Increased telomere replication efficiency	VUS	Not reported	Not reported	[40,69,151,152,153,162,163]
		rs1265164	Intronic regulatory	CST complex regulator	Regulation of telomere length via transcriptional mechanisms	Benign	~84%	~80%	[40,69,151,152,153,164]
		rs111447985	Intronic regulatory	CST complex regulator	Improved telomere replication stability	VUS	Not reported	Not reported	[40,69,151,152,153]

## 6. Research Gaps and Future Directions

Despite advances in the study of the biological mechanisms of telomeres and their role in human diseases, significant knowledge gaps remain, especially in underrepresented populations, such as Latin American populations [165]. Latin American populations are characterized by complex admixture involving Native American, European, and African ancestries, which can influence locus-specific effect sizes, variant penetrance, and overall polygenic risk score (PRS) study performance [28].

Current knowledge of genetic variants associated with telomere length (TL) is largely derived from European and Asian populations, which limits its generalizability and may overlook population-specific variants resulting from evolutionary, environmental, and demographic processes. Addressing this gap is essential to identify new genetic determinants of TL and better understand disease susceptibility in these populations [166]. Furthermore, given the highly polygenic architecture of TL regulation [22,25,68], PRS provide a potential framework for aggregating the effects of multiple genetic variants into a composite estimate of genetically determined TL [107,131,132,167]. However, the application of TL-PRS in Latin American populations has been limited, given that most TL-associated GWAS have been conducted in European populations [69,107,109].

Similarly, another relevant gap is the limited incorporation of TL measurements in population-based studies in Latin America. Expanding these efforts by including standardized TL measurements alongside genomic and phenotypic data would enable longitudinal analyses, facilitate biomarker discovery, and strengthen epidemiological studies on aging and cancer risk [69].

Therefore, to address these challenges, several strategies should be considered, including the development of population-specific GWAS, ancestry-aware modeling approaches, and the integration of environmental, lifestyle, and socioeconomic factors that influence telomere dynamics. This gap has direct scientific and healthcare implications, as studies in European-ancestry populations have generated actionable insights into TL-associated disease risk, prognosis, telomere biology disorders, cancer susceptibility, and potential risk-stratification tools. However, the limited representation of Latin American populations restricts our understanding of ancestry-specific allele frequencies, linkage disequilibrium patterns, variant effect sizes, and gene–environment interactions that may shape telomere biology in admixed populations. Consequently, diagnostic, prognostic, and preventive tools derived from non-Latin cohorts may have uncertain accuracy and limited clinical utility in Latin American settings. Interdisciplinary studies linking TL-associated variants, standardized TL measurements, environmental exposures, and clinical registries are therefore essential to support equitable translation into risk stratification, early diagnosis, prognosis assessment, and personalized interventions.

The present article is a narrative review, and the literature was selected to provide a broad synthesis of telomere biology, genetic variation, and disease associations, rather than following a predefined systematic search protocol. No formal database search strategy, predefined inclusion and exclusion criteria, or risk-of-bias evaluation was applied, as would be required for a systematic review or meta-analysis.

## Data Availability

No new data were created or analyzed in this study. Data sharing is not applicable to this article.
